# Intermediate-Term Outcomes of Dual Adult versus Single-Kidney Transplantation: Evolution of a Surgical Technique

**DOI:** 10.1155/2016/2586761

**Published:** 2016-07-10

**Authors:** Ana K. Islam, Richard J. Knight, Wesley A. Mayer, Adam B. Hollander, Samir Patel, Larry D. Teeter, Edward A. Graviss, Ashish Saharia, Hemangshu Podder, Emad H. Asham, A. Osama Gaber

**Affiliations:** ^1^Department of Surgery, Houston Methodist Hospital, Houston, TX 77030, USA; ^2^Department of Urology, Houston Methodist Hospital, Houston, TX 77030, USA; ^3^Department of Pharmacy, Houston Methodist Hospital, Houston, TX 77030, USA; ^4^Department of Pathology & Genomic Medicine, Houston Methodist Hospital, Houston, TX 77030, USA

## Abstract

*Background*. Acceptance of dual kidney transplantation (DKT) has proven difficult, due to surgical complexity and concerns regarding long-term outcomes. We herein present a standard technique for ipsilateral DKT and compare outcomes to single-kidney transplant (SKT) recipients.* Methods*. A retrospective single-center comparison of DKT and SKT performed between February 2007 and July 2013.* Results*. Of 516 deceased donor kidney transplants, 29 were DKT and 487 were SKT. Mean follow-up was 43 ± 67 months. DKT recipients were older and more likely than SKT recipients to receive an extended criteria graft (*p* < 0.001). For DKT versus SKT, the rates of delayed graft function (10.3 versus 9.2%) and acute rejection (20.7 versus 22.4%) were equivalent (*p* = ns). A higher than expected urologic complication rate in the DKT cohort (14 versus 2%, *p* < 0.01) was reduced through modification of the ureteral anastomosis. Graft survival was equivalent between DKT and SKT groups (*p* = ns) with actuarial 3-year DKT patient and graft survivals of 100% and 93%. At 3 years, the groups had similar renal function (*p* = ns).* Conclusions*. By utilizing extended criteria donor organs as DKT, the donor pool was enlarged while providing excellent patient and graft survival. The DKT urologic complication rate was reduced by modification of the ureteral anastomosis.

## 1. Introduction

Despite increasing wait times for deceased donor transplant candidates, a significant percentage of procured kidneys are not utilized because they are perceived to have inadequate function. According to Scientific Registry of Transplant Recipients (SRTR) data, almost half of extended criteria donor kidneys and one-third of kidneys with serum creatinine greater than 1.5 mg/dL are currently discarded [1]. It is likely that clinicians declined to utilize these organs out of a concern that inadequate functional reserve would result in suboptimal function and early graft loss. In order to address this problem, dual adult kidney transplantation (DKT) has proven a useful option for utilization of marginal kidneys [2]. Furthermore, a number of reports have demonstrated comparable graft survival between DKT from marginal donors to single-kidney transplants utilizing both expanded and standard criteria donors [3–6].

Widespread implementation of DKT has however proven difficult, likely due to the complexity of the surgical procedure and concerns regarding poor outcomes. Moreover, while most groups [3, 6–9] have advocated for ipsilateral placement of both donor kidneys in order to reduce operating time, there is a paucity of data describing the operative technique. Through a review of our own experience with DKT, we sought to present a standard technique for performing ipsilateral DKT and to compare intermediate-term outcomes of DKT to that of single-kidney transplant recipients (SKT).

## 2. Methods

### 2.1. Patient Population

This is a single-center retrospective review of all deceased donor kidney transplants performed between February 2007 and July 2013, with particular attention to dual kidney transplants (DKT). Multiorgan and pediatric en bloc transplants were excluded.

### 2.2. Selection Criteria for Dual Kidney Transplantation

Donor organs for DKT and single-kidney transplant (SKT) recipients were procured from both donation after brain death (DBD) and donation after cardiac death (DCD) donors.

Two categories of kidneys were used for dual transplantation. The first category comprised kidneys from expanded criteria donors (ECD), defined as deceased donors (1) greater than 60 years old or (2) greater than 50 years old and with at least 2 of the following criteria: (a) a history of hypertension, (b) terminal serum creatinine greater than 1.5 mg/dL, or (c) death due to a cerebrovascular accident. The second category consisted of kidneys from standard criteria donors (SCD) that were deemed functionally compromised due to high serum creatinine, poor pump characteristics, or unfavorable histology on biopsy.

All donor kidneys utilized for DKT met the UNOS criteria for dual kidney allocation with at least 2 of the following criteria: (1) age > 60 years, (2) eGFR < 65 mL/min/1.73 m^2^, (3) Cr > 2.5 mg/dL, (4) history of longstanding diabetes or hypertension, or (5) glomerulosclerosis between 15 and 50% [10]. All kidneys had previously been turned down by other local transplant centers for use as single-kidney transplants due to the above noted donor characteristics. After procurement, all kidneys were preserved by pulsatile hypothermic perfusion.

Kidneys were rejected for DKT if (1) greater than 25% of glomeruli were sclerotic on procurement biopsy, (2) estimated GFR at procurement was less than 50 mLs/min, or (3) at initial gross inspection there were multiple cysts or extensive atherosclerosis extending into the renal arteries.

### 2.3. Recipient Selection for DKT

Recipients selected to receive a DKT were matched by donor age and excluded recipients with a body mass index (BMI) greater than 35 or less than 22. All recipients had previously consented to receive an ECD kidney. All patients receiving dual kidney transplants were informed of the risks and benefits of the procedure.

The Houston Methodist Research Institute Institutional Review Board approved this retrospective review.

### 2.4. DKT Surgical Procedure

One surgical team performed all transplants. The decision to proceed with DKT was made after standard bench preparation of the donor kidneys. A curvilinear incision extending from the symphysis pubis to the anterior superior iliac crest was utilized, similar to but longer in length than the incision used for single-kidney transplantation. This permitted complete dissection of the common internal and external iliac vessels in the extraperitoneal space. The right donor kidney was placed in the superior position. The vein-to-vein anastomosis was performed between the renal vein and the lower cava or common iliac vein. The proximal common iliac artery was used for the end to side arterial anastomosis. For the inferior-positioned donor kidney, the end to side venous anastomosis and end to side arterial anastomoses were performed between the donor vessels and the external iliac vein and artery of the recipient, respectively.

Although the vascular anastomoses were standardized, the ureteral anastomoses evolved to address a higher than expected incidence of ureteral stricture. Initially, both ureters were anastomosed to the bladder using the standard Lich-Gregoir technique, as previously described [9]. Over the course of the series, the ureteral anastomosis technique was revised. Initially, the native ureter was anastomosed to the upper transplant kidney ureter as an end-to-end ureteroureterostomy. Subsequently, we further modified the technique in order to anastomose the native ureter to the upper transplant kidney pelvis as ureteropyelostomy. The lower transplant kidney ureter was anastomosed directly to the bladder using the standard technique. All ureteral anastomoses were performed over ureteral stents (6 French Greene Renal Transplant Stent Set, Cook Medical, Indiana, USA).

### 2.5. Immunosuppression

Recipients of DKT and SKT were treated with the same immunosuppression protocol. Subjects considered at high risk of acute rejection (African Americans (AA) recipients, retransplants, and recipients with PRA > 20%) received a 3-day course of rabbit antithymocyte globulin (rATG) at a dose of 1.5 mg/kg/day. All other subjects received either 2.0 mg/kg of daclizumab or 20 mg of basiliximab for 2 doses. Maintenance immunosuppression consisted of tacrolimus, mycophenolate mofetil (MMF), and prednisone. The dose of tacrolimus was adjusted to maintain a trough level of 8–10 ng/mL for the first 3 months after transplantation and tapered to 5–8 ng/mL thereafter. MMF was given at a dose of 1000 mg twice daily. Methylprednisolone (250 mg) was given on the day of transplantation, tapered to 25 mg by day 5 and then to 5–10 mg by 6 months after transplantation.

### 2.6. Determination of Posttransplant Donor Specific Antibody

Patient sera were tested for the presence of de novo donor specific antibody (dnDSA) using a multiplex solid phase bead array (LABScreen; One Lambda/OLI, Canoga Park, CA) on a Luminex cytometer (Luminex 100, Luminex, Austin, TX). Data were analyzed using Fusion software (LABScreen; One Lambda/OLI, Canoga Park, CA), and the results were recorded as the mean intensity fluorescence (MFI). All beads showing an MFI > 2000 were considered positive. De novo DSA were defined as antibodies directed against the donor HLA that were not present prior to transplant and included HLA A, B, CW, DR, DR51/52/53, DQ, and DP. DSA were tested at 1, 2, 3, 6, 9, and 12 months after transplant or for clinical indication.

### 2.7. Determination of BK Viremia

BK virus testing by polymerase chain reaction (PCR) was performed at 1, 3, 6, 9, 12, 18, and 24 months after transplant or for clinical indication. A viral load of greater than 300 viral copies/mL was considered positive.

### 2.8. Diagnosis of Acute Rejection

Renal biopsies were evaluated by light microscopy and immunofluorescence. Electron microscopy examination was performed when glomerular pathology was suspected. Histopathology and classification of rejection were reported according to the Banff 2005 Classification of Renal Allograft Pathology and subsequent updates [11].

### 2.9. Statistical Analysis

Categorical characteristics of kidney transplant recipients who received a dual kidney transplant were compared to those with received a single-kidney transplant by Chi square or Fisher's exact tests, where appropriate. Logistic regression was used to analyze continuous covariates. Graft survival and acute rejection were visualized using Kaplan Meier statistics with significance assessed by log rank test. *p* values < 0.05 were considered statistically significant. Statistical analyses were conducted with Stata SE version 13.1 (StataCorp, College Station, TX, USA) and SAS 9.3 (SAS Institute Inc., Cary, NC, USA).

## 3. Results

516 deceased donor kidney transplants were performed at our institution between February 2007 and July 2013, of which 29 were DKT and 487 were SKT. Mean follow-up after transplant was 43.3 ± 65.6 months.

### 3.1. Baseline Characteristics

Donor and recipient data for DKT and SKT recipients are summarized in Table 1.

Donors for DKT were older and more likely to have had a history of diabetes and hypertension, compared to donors for SKT (*p* < 0.001). Similarly, DKT recipients were older than SKT recipients, less likely to be highly sensitized, and more likely to have diabetes as the cause of their end stage renal disease (*p* < 0.05).

### 3.2. Expanded Criteria and Donation after Cardiac Death Donors

In the DKT group, 17 of the 29 (58.6%) donors met criteria for ECD classification and 3 donor organs (10.3%) were procured after cardiac death (DCD). Two donors met both ECD and DCD criteria. The use of 12 non-ECD donors for DKT was based principally on a low estimated GFR in 6, unfavorable histology in 4, and poor pump parameters in 2 cases. In the SKT group of 487 patients, 68 (14.0%) organs were from ECD donors, and 46 (9.4%) were procured from DCD donors. No SKT organs met criteria as both ECD and DCD donor. DKT recipients were more likely than SKT recipients to receive an ECD organ (*p* < 0.001); however there was no difference between the groups with regard to receipt of DCD organs (*p* = ns).

### 3.3. Post-Operative Outcomes

The average hospital length of stay after transplant for DKT recipients was 6 days, versus 3 days for SKT recipients. Three of 29 DKT recipients (10.3%) suffered delayed graft function (DGF) versus 45 of 487 SKT patients (9.2%, *p* = ns). The incidence of acute rejection was 20.7% (*n* = 6) in the DKT group versus 22.4% (*n* = 109) in the SKT group (*p* = ns). The incidence of dnDSA was slightly higher in the DKT group, 48.3% (*n* = 14) versus 31.6% (*n* = 154) of the SKT recipients (*p* = ns). The incidence of BK viremia was 27.6% (*n* = 8) in the DKT group, versus 22.0% (*n* = 107) in the SKT group (*p* = ns).

### 3.4. Urological Complications

Within the first year after transplantation, 4 of 29 DKT patients (14%) developed urologic complication, compared to 10/487 SKT recipients (2%, *p* < 0.01). All of the urologic complications in the DKT group were due to ureteral strictures, generally occurring at the ureter to bladder anastomosis. Moreover, 3 of the 4 complications occurred in the upper kidney only, and the fourth involved both kidneys. Within the SKT group, 6 complications were due to anastomotic strictures and 4 were caused by urine leaks.

**Figure 1:**
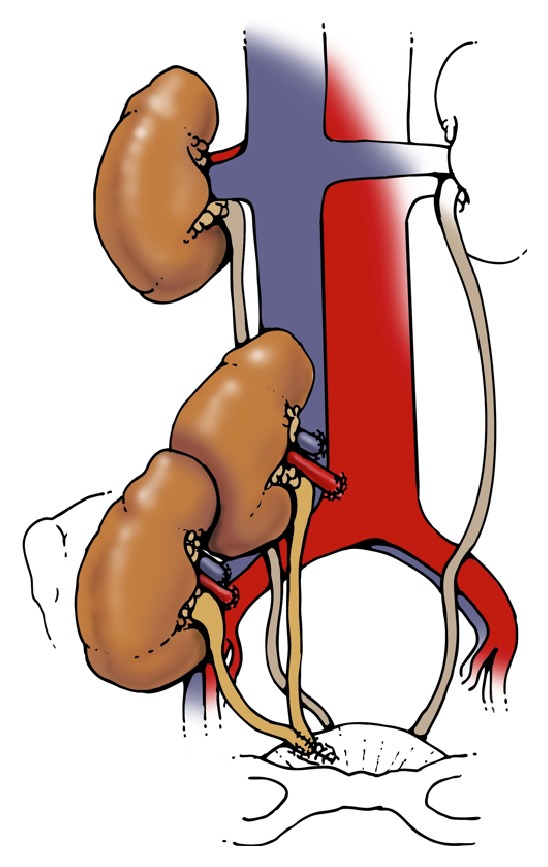
Ureteroneocystostomy.

**Figure 2:**
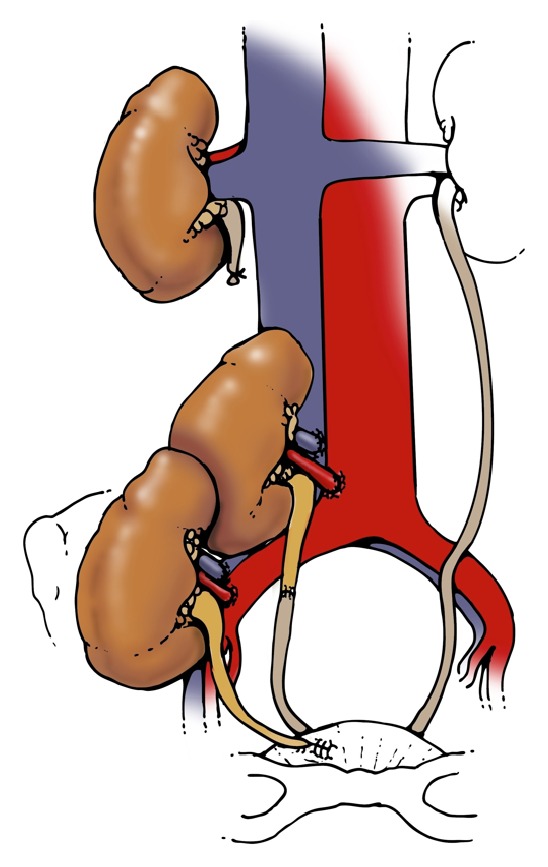
Ureteroureterostomy.

**Figure 3:**
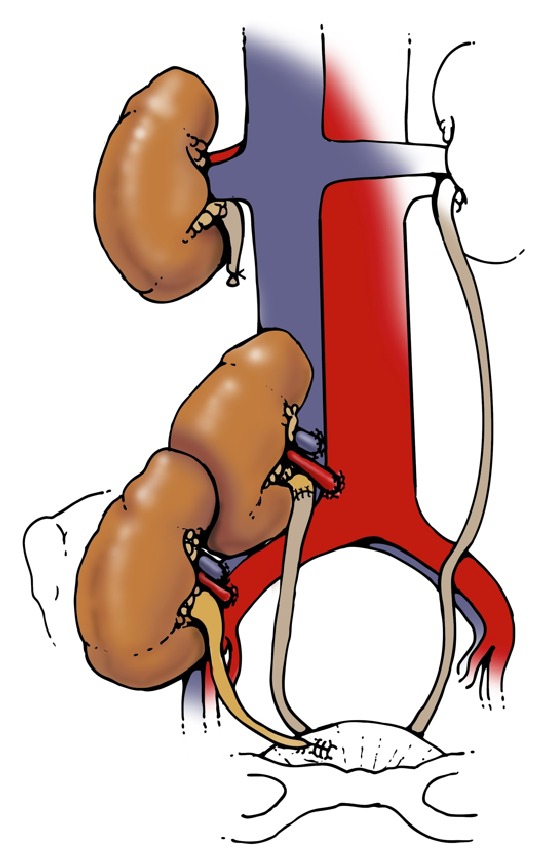
Ureteropyelostomy.

To understand the reason for the high incidence of ureteral complications in the DKT group, we examined the surgical technique in all procedures. Three of 4 recipients with ureteral obstruction had ureteral anastomoses performed via the original Lich-Gregoir technique, with both transplant ureters anastomosed to each other prior to insertion into the bladder (Figure 1). Of the 10 transplants performed in this fashion, 3 resulted in obstruction, for a 30% complication rate. In contrast, when the native ureter was utilized and the upper transplant ureter shortened in the modified technique (Figures 2 and 3), only one in 19 transplants (5%) suffered ureteral complication.

### 3.5. Graft Survival and Renal Function

**Figure 4:**
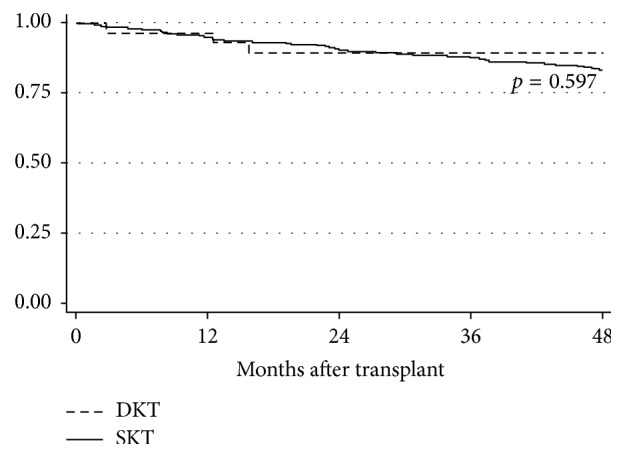
Actuarial graft survival of dual kidney transplant (DKT) and single-kidney transplant (SKT) recipients.

There was no difference in graft survival between the DKT and SKT groups, as shown in Figure 4 (*p* = ns). Specifically, for the DKT group, actuarial patient and graft survivals at 3 years were 100% and 93%, respectively. There were four graft losses in total. One graft was lost at two months after transplant because of recurrent hemolytic uremic syndrome. Another graft loss at 6 months was multifactorial, likely failing due to the combined effects of donor disease, BK viremia, and rejection. A third graft loss at 15 months after transplant was caused by patient noncompliance with immunosuppression therapy, and the fourth graft loss at 5.5 years after transplant was due to chronic allograft nephropathy. There were no patient deaths in this cohort.

The mean serum creatinine and estimated glomerular filtration rates (GFR, using the MDRD equation) of both DKT and SKT groups are summarized in Table 2. The groups had comparable renal function at each time point, with the exception of slightly better renal function in the DKT group in the first month after transplant (*p* = 0.001).

## 4. Discussion

In this single-center experience, we found that up to 4-years after transplantation, actuarial DKT patient and graft survivals were equivalent to those of SKT. Moreover, despite a greater percentage of ECD kidneys among the DKT cohort, the incidence of DGF, acute rejection, DSA, and BK viremia were also similar between the 2 groups. Importantly, the quality of the donor kidney function was equivalent at all time points studied.

There were more urologic complications in the DKT cohort, for the most part due to a greater likelihood of developing a stenosis at the ureter to bladder anastomosis. In this series, most urologic complications occurred with the initial technique (side to side anastomosis of the two ureters and single tunneled ureteroneocystostomy). This was likely due to the fact that the upper transplant kidney ureter was too long. Given the segmental periureteric vascular plexus, the distal ureter may have become compromised due to a limited blood supply, leading to ischemia, stricture, and obstruction. By shortening the length of the upper transplant ureter and using the native ureter, the complication rate declined. But given the small number of cases performed using the revised technique it remains speculative as to whether this problem has been solved. Yet, this technique has been described in previous publications. Wu et al. published a single-case report regarding ipsilateral placement of dual kidneys in a patient who underwent simultaneous nephrectomy for polycystic kidney disease. Similar to our modified technique, a Lich-Gregoir anastomosis was used for the lower kidney, and ureteroureterostomy was formed between the right native ureter and the upper transplant ureter [12]. In another series of 24 ipsilateral DKT, the ureteral anastomosis was created in one of the three ways: (1) a single conjoined ureteroneocystostomy, (2) implanted via separate ureteroneocystostomies, or (3) separate ureteroureterostomies between the transplant and native ureters. In this report, all the 3 ureteral complications (one fistula and 2 strictures) occurred when the two transplant ureters were conjoined [13]. An alternative explanation for the higher urologic complication rate in our series may be that the conjoined technique resulted in ischemic injury to the distal ureter that could be avoided by separate implantation of the ureters directly into the bladder. This is the technique most commonly used in other large series and has been associated with a low rate of complications.

Our data is generally consistent with previous published reports [2–6] of dual kidney transplantation processes. The largest series of DKT belongs to that of Rigotti et al. [3] who published their experience with 200 cases. Seventy-five percent of transplants were placed unilaterally and 5-year graft survivals were equivalent to that of single-kidney transplants. They did not compare the incidence of ureteral complications between DKT and SKT, although the overall incidence of such events was relatively low. In an earlier publication they had compared their experience of 100 ipsilateral DKT with a cohort of single-kidney transplants performed using the same selection criteria and showed comparable surgical complication rates. Reported complications included stenosis of the ureteroneocystostomy anastomosis, yet there was no difference in incidence between the two groups [14]. A multicenter experience reported by Nardo et al. [4] analyzed 80 dual kidney transplants from ECD donors and compared outcomes to those of recipients of single-kidney transplantations from both expanded criteria and standard criteria donors. The DKT were performed in either an ipsilateral or bilateral fashion. They noted a 14% incidence of ureteral complications, with 8/11 requiring surgical reexploration. Yet, the frequency of urological complications did not differ among the different groups.

Our series is novel in two significant ways. Firstly, we did not exclusively utilize ECD kidneys for our DKT, as a number of the kidneys were from donors less than 50 years old with compromised function that would otherwise have been discarded. This is an important point, as most reports of dual kidney transplantation have focused on utilizing exclusively ECD donors or those with reduced GFR. In our series we expanded our acceptance criteria to include any donor with impaired renal function, regardless of age. In this way, we were able to successfully utilize organs from younger donors with biopsy criteria or pump perfusion parameters that resulted in their rejection for use as single kidneys. Secondly, few reports have focused on surgical technique, specifically ureteral complications. The barrier to greater use of this technique remains a technical one: the operating time is longer, the caval anastomosis is more challenging, and potential for ureteral complications is higher. This report provides a detailed description of the surgical procedure and potential complications.

In summary, by utilizing marginal quality donor organs as DKT, we have increased the available donor pool while providing excellent function, patient, and graft survival. Ureteral complications may be avoided by shortening the length of the upper transplant kidney ureter. Standardizing this surgical technique resulted in excellent outcomes, providing a means to utilize marginal donor kidneys and address the problem of donor organ shortage.

## Figures and Tables

**Table 1:** Pretransplant demographic data for dual (DKT) and single-kidney transplant (SKT) recipients.

	DKT, *n* = 29	SKT, *n* = 487	*p* value
Donor age, years, median (IQR)	58 (55–66)	39 (23–50)	<0.001

Donor male gender	13 (44.8%)	193 (39.6%)	0.475

Donor race			
(i) Caucasian	16 (55.2%)	259 (53.2%)	0.835
(ii) African American	5 (17.2%)	78 (16.0%)	0.797
(iii) Hispanic	5 (17.2%)	137 (28.1%)	0.284

Donor terminal Cr, mg/dL, median (IQR)	1.1 (0.8–1.5)	1 (0.8–1.4)	0.301

Donor comorbidities			
(i) Hypertension	18 (62.1%)	114 (23.4%)	<0.001
(ii) Diabetes	11 (37.9%)	24 (4.9%)	<0.001

Cold ischemia time, hours, median (IQR)	20 (17–26)	20 (15–26)	0.547

Recipient age, years, median (IQR)	61 (56–67)	51 (41–61)	<0.001

Recipient male gender	19 (65.5%)	279 (57.3%)	0.384

Recipient race			
(i) Caucasian	9 (31.0%)	153 (31.4%)	0.966
(ii) African American	9 (31.0%)	168 (34.5%)	0.703
(iii) Hispanic	5 (17.2%)	126 (25.9%)	0.383

Recipient indication for transplant			
(i) Hypertension	11 (37.9%)	216 (44.4%)	0.498
(ii) Diabetes	10 (34.5%)	88 (18.1%)	0.029

Recipient time on dialysis, years, median (IQR)	2.5 (1.25–3.5)	3 (1.5–5)	0.101

Recipient dialysis type			
(i) Hemodialysis	16 (55.2%)	323 (66.3%)	0.303
(ii) Peritoneal dialysis	4 (13.8%)	68 (14.0%)	
(iii) Preemptive (no dialysis)	9 (31.0%)	96 (19.7%)	

Recipient PRA ≥ 20%	6 (20.7%)	252 (51.7%)	0.001

**Table 2:** Posttransplant renal function for dual (DKT) and single-kidney transplant (SKT) recipients.

Time	DKT		SKT		*p* value
	Median SCr (IQR) in mg/dL	Median eGFR (IQR) in mL/min/1.73 m^2^	Median SCr (IQR) in mg/dL	Median eGFR (IQR) in mL/min/1.73 m^2^	
1 month	1.1 (0.8–1.5)	66.4 (46.4–89.0)	1.3 (1.0–1.7)	54.7 (42.7–68.5)	0.001
6 months	1.3 (0.9–1.8)	47.5 (39.7–74.5)	1.2 (1.0–1.6)	58.2 (47.7–70.5)	0.619
12 months	1.4 (1.0–1.7)	56.0 (42.6–67.9)	1.2 (1.0–1.6)	58.4 (46.4–73.0)	0.257
18 months	1.2 (0.9–1.6)	60.2 (46.4–77.9)	1.2 (1.0–1.6)	60.6 (46.6–74.2)	0.358
24 months	1.3 (0.9–1.8)	53.4 (46.4–66.4)	1.2 (1.0–1.6)	58.5 (45.1–74.6)	0.873
36 months	1.6 (1.0–2.1)	45.9 (36.8–62.6)	1.3 (1.0–1.7)	56.7 (43.7–71.8)	0.952
